# A study of empathy levels among nursing interns: a cross-sectional study

**DOI:** 10.1186/s12912-023-01381-y

**Published:** 2023-06-30

**Authors:** Suaad Ghazwani, Amira Alshowkan, Nagla AlSalah

**Affiliations:** 1 Master of Psychiatric and Mental Health Nursing, College of Nursing, Imam Abdulrahman Bin Faisal University, Dammam, Saudi Arabia; 2 Department of Community Nursing, College of Nursing, Imam Abdulrahman Bin Faisal University, Dammam, Saudi Arabia

**Keywords:** Empathy, Interpersonal reactivity index, Nursing Interns, Quantitative study

## Abstract

**Background:**

Empathy is one of the therapeutic communication techniques used to help the client feel better. However, there are a few studies have investigated level of empathy among enrollers at nursing colleges. The aim was to examine the level of self-reported empathy among nursing interns.

**Methods:**

The study was a descriptive, cross-sectional in nature. A total of 135 nursing interns fill in the Interpersonal Reactivity Index from August to October 2022. Data was analyzed through the SPSS program. An independent –sample t-test and one way- ANOVA was used to explore differences in the degree of empathy with respect to academic and sociodemographic factors.

**Results:**

The results of this study showed that nursing interns showed a mean level of empathy of 67.46 (SD = 18.86). This result indicated that the nursing interns have moderate levels of empathy overall. There was statistical significant difference in the mean level of subscales of perspective-taking and empathic concern between males and females. Additionally, nursing interns who are less than 23 years old scored high in the subscale of perspective-taking. Married nursing interns and who preferred nursing as a profession scored higher in the subscale of empathic concern than unmarried ones and who did not preferred nursing as a profession.

**Conclusion:**

Perspective taking incresed with younger male nursing interns, this reflects high cognitive flexibility with younger age nursing interns. Morover, the empathic concern incresed with male married nuring interns who preferred nursing as a profession. This implies that they should engage in continuous reflection and educational activities as part of their clinical training as nursing interns in order to improve their empathic attitudes.

**Supplementary Information:**

The online version contains supplementary material available at 10.1186/s12912-023-01381-y.

## Background

The term “empathy” first emerged in the field of psychology as a result of Titchener’s translation of the German word “einfühlung,“ which literally means “feeling into,“ or the ability to understand another person’s feelings [1]. Empathy has been researched in a variety of domains of psychology, including developmental psychology [2], social psychology [3], and forensic psychology [4], these domains cover a wide range of themes, including relationships and marriage, violence and sexual offenses, early development, and autism. There is a growing focus in empathy in relation to management and marketing in occupational psychology [5, 6], and empathic processes in client and colleague relationships [7]. Empathy studies in healthcare has likewise spanned 50 years, with early studies conducted by Aring [8] in medicine, Peplau [9] in nursing, and Rogers [10] in counselling.

One of the characteristic of a good nurse is empathy in patient care [11]. Patients benefit from the use of empathy as a part of therapeutic communication. It is the capacity to comprehend and recognize the reality of another individual, as well as to effectively recognize and convey one’s own feelings to others [12]. Empathy is an expression of understanding what it is like for a distressed, suffering, or upset client [13]. According to Davis [14], empathy encompasses both cognitive and affective or emotional domains. The cognitive domain refers to one’s capability to comprehend another’s inner thoughts and feelings. It’s also the ability to see the world through the eyes of another person [15]. The affective domain, instead, encompasses the ability to enter or take part another person’s emotional experience. Empathy and sympathy are not the same thing. These are frequently combined into a single terminological category. A sympathetic nurse shares their own feelings with the patient, whereas an empathetic nurse shares the patient’s thoughts [16].

A number of characteristics have been linked to the nurses’ empathetic behavior. Nurses who are empathetic and concerned about their patients and who communicate effectively with them are more likely to provide the highest quality of care [17]. Empathy is still taught in nursing programs due to previous characteristics; undergraduate nursing students are learning the significance of creating an empathic relationship with patients as well as fundamentals communication skills [18]. Nursing students with a higher level of empathy had a more positive attitude toward elderly patients [19]. Nursing students who were more empathic were better at taking histories and performing health assessments [20]. They were satisfied, and their therapeutic relationship with the patients was better [21].

Nursing students complete an internship year during their final year of nursing education [22]. Internships are available for students after fourth year of bachelor education who have accomplished their formal education up to that point. It is a transitional time. Nursing Interns earn more clinical skills through this time because there is less lecture room instruction and more clinical skills [22]. Nursing interns work around the clock in psychiatry wards, emergency departments, operating room, primary health clinics, labor rooms, and other clinical areas. These nursing interns are expected to pay attention to the patients’ needs and desires and to develop therapeutic relationships with them in order to better meet patients’ expectations. As a result, internship is a time of significant personal and professional transformation. Longer work hours, increased patient care demands, and less time for family are just a few of the changes that have occurred.

Empathy plays a crucial role in nurse-patient encounters as well as clinical outcomes; for this reason, nursing education and nursing internship programs should make it a priority in their curriculum [13, 23]. However, the nursing curriculum did not provide teaching empathy skills as a separate course or in the form of a preparation workshop for nursing students or interns in nursing schools in Saudi Arabia. Instead, the nursing curriculum integrated these skills into broader courses on patient care, psychology, and professional ethics [24].

Studying the varying empathy levels among nursing interns could provide insight into their educational paths, which is relevant to nursing education and clinical practice. Empathy is linked to a reduction in clinical practice stress and burnout among nursing interns [25]. Song [26] found that reducing personal distress of empathy is important for nursing interns, and that it may help to reduce clinical practice stress and burnout. Identifying the level of empathy in nursing interns at risk of stress and anxiety may assist curriculum developers in effectively integrating interventions to promote empathy levels in nursing interns. Identifying nursing interns at risk of having a low level of empathy can also help professionals predict complications and use appropriate intervention. As a result, the aim of the present study is to examine the level of self-reported empathy among nursing interns as well as the factors influencing them during their internship.

## Methods

### Research design

The study aim is addressed using a descriptive, cross-sectional design.

### Study setting

The study was carried out at a college of nursing at Imam Abdulrahman Bin Faisal University in Saudi Arabia Study from August to October 2022. The Bachelor of Basic Science in nursing program consists of four years of formal education and one year of internship. Following successful completion of the undergraduate nursing program’s four years, the student must complete a 52-week hospital-based internship in recognized hospitals that can provide a proper training area to meet the internship program’s objectives [27].

### Sample description

The study’s target population was nursing interns who had completed four years of study and have begun their 52-week internship training period. A non-probability convenience sampling method was used to recruit nursing interns. A convenience sample obtained by approaching the subjects who are available at the time of data collection until the required sample size reached.

### Inclusion and exclusion criteria

The selection and inclusion criteria were required that the nursing interns (a) who are willing to take part in the study, (b) who successfully completed the four-year undergraduate nursing program at IAU’s college of nursing, (c) who have completed the orientation program (2 months) and are now in the clinical setting). Nursing interns who were still enrolled in the orientation program at the IAU’s college of nursing were excluded.

### Sample size

The sample size was calculated prior to data collection using the G*power software version 3.1 [28].The researcher estimates the sample size on the basis of a desired power = 0.80, a present α = 0.05 and medium effect size (0. 5). The total sample consists of 128 participants to strengthen the study findings. However, over sampling is intended to gain more understanding of the phenomena. The final sample size in this study was 135 nursing interns.

### Study instruments

#### Interpersonal reactivity index (IRI)

The IRI (Davis [14] is a 28-item scale with four, seven-item subscales: the Fantasy Scale (FS), Perspective Taking Scale (PT), the Empathic Concern Scale (EC), and the personal distress scale (PD). Compassion and concern for others are referred to as EC. Perspective Taking Scale (PT) evaluates unintentional attempts to adopt other people’s viewpoints. The Fantasy Scale (FS) stands for the probability that a person will identify with an imaginary character. When exposed to the negative experiences of others, PD indicates the degree to which an individual feels uneasy or worried. Each item is rated on a Likert-like scale of 5-point ranging from 0 = does not describe me well to 4 = describes me very well. The possible overall score ranges from 0 (very low level of empathy) to 112 (very high level of empathy), with a mean score of 56. This suggests that nursing interns with higher mean scores are generally geared toward a high level of empathy [14]. The aggregate scores for each sub-dimension are calculated by averaging the responses to each seven-item subscale.

The ISI produced moderate internal consistency ranging from.70 to.78 in Davis’s original report (Davis, 1994). Internal consistency of the subscales was moderate (FS = 0.78; PT = 0.75; EC = 0.71; PD = 0.78). Other previous studies report Cronbach’s alpha values ranging from 0.70 to 0.83 for IRI subscales and correlation coefficients ranging from 0.01 to 0.37 between subscales [29–31]. A pilot study to evaluate the readability and clarity of the instruments was conducted using a convenient sample of 20 nursing interns who had characteristics similar to those of the study subjects. All participants reported that the wording of the instruments and the instructions were clear. Cronbach’s alpha was 0.89 in the current study, indicating that IRI has very good internal consistency.

#### Sociodemographic and educational questionnaire

The researchers created a demographic questionnaire to collect demographic and educational characteristics. Sociodemographic characteristics included gender, marital status, and age. Academic characteristics included nursing intern’s preference of nursing as profession, clinical training area, and Grade Point Average (GPA). The questions were derived from previous reviewed studies in similar study samples [16, 32] .

### Ethical considerations

The current study was approved by Imam Abdulrahman Bin Faisal University’s (IAU) Institutional Review Board (Ref. No. IRB-PGS-2022-04-164). Furthermore, the author notified the dean of nursing and the nursing internship coordinator. Nursing interns were persuaded that completing the questionnaires would have no effect on their academic status and progress within the college of nursing. Nursing interns were notified that involvement in the study was completely optional, and their privacy and confidentiality would be respected. Consent form was secured from study’s participants.

### Data analysis

The Statistical Package for Social Science (SPSS) SPSS®-PC version 28 For Windows was used to analyze the data. For all statistical analysis, the level of significance was set at 0.05. Descriptive statistics were used to define the socio-demographic features of the sample as well as the instrument of IRI. An independent –sample t-test was used to examine difference in the level of empathy relation to gender, marital status, and preferred nursing as a profession. Furthermore, one-way analysis of variance (One way- ANOVA) was used to explore differences in the degree of empathy with respect to the age groups, GPA, and clinical training area.

## Results

### Sociodemographic characteristics of participants

A total of 135 nursing interns participated in this study with a response rate of 100%. Majority of participants were female (64.4%), unmarried (74.8%) and age ranged from 21 to 26 years, with a mean (SD) of 23.10 (1.05). Most of the participants (86.7%) prefer nursing as a profession. More than half of nursing interns (55.6%) had > 4.1 GPA points, with a mean (SD) of 4.06 (0.51). There were several training areas for nursing interns at the time of data collection, but the most common training location was the emergency department (17.8%) (Table 1).

**Table 1:** Socio-demographic Characteristics of Nursing Interns, N = 135. SD = standard deviation

Variable	Mean	SD	NO (%)
**Age**	23.10	1.05	
**Grade point average (GPA)**	4.06	0.51	
**Age Groups**			
≤ 23 >24			93(68.9) 42(31.1)
**Grade point average (GPA)**			
≤ 3 3.1-4 > 4.1			9(6.7) 51(37.8) 75(55.6)
**Gender**			
Female Male			87(64.4) 48(35.6)
**Marital Status**			
Unmarried Married			101(74.8) 34(25.2)
**Preferred nursing as a profession**			
Yes No			117(86.7) 18(13.3)
**Training Area**			
Medical Ward Surgical Ward Emergency Department Catheterization Lab. Gynaecology Ward Coronary Care Unit (CCU) Operative Room Hemodialysis Intensive Care Unit (ICU) Pediatric Ward Outpatient Department (OPD) Endoscopy Ward Orthopedic Ward			21(15.6) 14 (10.4) 24 (17.8) 9 (6.7) 5 (3.7) 9 (6.7) 11 (8.1) 10 (7.4) 13 (9.6) 6 (4.4) 10 (7.4) 2 (1.5) 1 (0.7)

### The results of nursing Interns’ self-reported Empathy Level

The mean level of total self-reported empathy that was measured by Interpersonal Reactivity Index (IRI) among nursing interns was 67.46 (SD = 18.86). The mean score is slightly above the midpoint of the IRI scale, which is 56, indicating that the nursing interns in the study may have moderate levels of empathy overall. Regarding to the subscales of IRI, Perspective-taking has the highest mean 17.7 (SD = 5.34) among nursing interns (Table 2). Therefore, the nursing interns in this study are capable of viewing a situation or comprehending a subject from a different perspective, such as that of another person.

**Table 2:** Measures of Central Tendency of Interpersonal Reactivity Index and the Subscales. SD = standard deviation

Variables	Minimum	Maximum	Mean	SD
Interpersonal Reactivity Index (IRI)	23	112	67.46	18.86
Fantasy scale (FS)	3	28	16.6	6.15
Perspective-taking scale (PT)	4	28	17.7	5.34
Empathic concern scale (EC)	4	28	16.7	4.73
Personal distress scale (PD)	1	28	16.3	5.65

### The results of relationship between level of self-reported Empathy, and Sociodemographic factors among nursing Interns

#### Gender

An independent –sample t-test was used to examine difference in the total level of empathy and subscales with respect to gender. The result of study showed that there is statistical significant difference in the mean level of perspective-taking subscale in scores between males (M = 19.21, SD = 5.31) and females (M = 16.95, SD = 5.21, t (133) = 2.38, p = 0.01) (Table 3). Moreover, there is statistical significant difference in the mean level of empathic concern subscale in scores between males (M = 18.04, SD = 5.04) and females (M = 16.07, SD = 4.43, t (133) = 2.35, p = 0.02) (Table 3). This means that male nursing interns are capable of viewing a situation or comprehending a subject from a different perspective, such as that of another person than female interns. Moreover, male nursing interns felt more sympathy and caring for others than female nursing interns. On the other hand, no statistical significant difference at the p < 0.05 between gender with total level of empathy (t (133) = 1.28, p = 0.20), and subscales of fantasy (t (133) = 0.58, p = 0.55), and personal distress (t (133) = − 0.50, p = 0.61). These results show that male and female nursing interns had the same total level of empathy and subscales of fantasy and personal distress.

**Table 3:** Difference between Gender and Level of IRI with Subscales, *Significant at p ≤ 0.05

Variable	Gender	Mean	SD	*t -Value*	*df*	*P-value*
Interpersonal Reactivity Index (IRI)	Male	70.27	20.24	1.28	133	0.200
	Female	65.91	17.99			
Fantasy	Male	17.02	6.30	0.58	133	0.557
	Female	16.37	6.09			
Perspective-taking	Male	19.21	5.31	2.38	133	0.018*
	Female	16.95	5.21			
Empathic concern	Male	18.04	5.04	2.35	133	0.020*
	Female	16.07	4.43			
Personal distress	Male	16.00	6.71	− 0.50	133	0.612
	Female	16.52	5.00			

#### Age

Pearson product –Moment correlation (Pearson’s r), coefficient for a 2-tailed test of significant was used to determine the relationship between total level of empathy and subscales with respect to age (Table 4). The result reveled that there is statistical significant negative relationship between the level of perspective-taking subscale and age in years (r = − 0.195; n = 135; p = 0.023). This means that as nursing intern age increases, the level of perspective-taking subscale decreases.

**Table 4:** Correlations between age and IRI with Subscales, *Significant at p ≤ 0.05

Variable	Age	*r*	*P-value*
Interpersonal Reactivity Index		− 0.030	0.734
Fantasy		0.008	0.924
Perspective-taking		− 0.195*	0.023
Empathic concern		− 0.060	0.492
Personal distress		0.127	0.142

An independent –sample t-test was used to examine difference in the total level of empathy and subscales with respect to age group. The result reveled that there is statistical significant difference in the mean level of perspective-taking subscale in scores between nursing interns less than 23 years (M = 18.61, SD = 4.87) and nursing interns more than 24 years old (M = 15.86, SD = 5.87, t (133) = 2.84, p = 0.005) (Table 5). These results reveled that nursing interns who are less than 23, scored higher in perspective-taking dimension more than nursing interns above 23 years. This means that younger nursing interns in this study are capable of viewing a situation or comprehending a subject from a different perspective, such as that of another person than older nursing interns.

**Table 5:** Difference between Age Group and IRI with Subscales

Variable	Age	Mean	SD	*t -Value*	*df*	*P-value*
Interpersonal Reactivity Index	≤ 23	69.30	17.90	1.70	133	0.092
	> 24	63.38	20.47			
Fantasy	≤ 23	17.11	5.96	1.43	133	0.155
	> 24	15.48	6.49			
Perspective-taking	≤ 23	18.61	4.87	2.84	133	0.005*
	> 24	15.86	5.87			
Empathic concern	≤ 23	17.24	4.72	1.71	133	0.089
	> 24	15.74	4.66			
Personal distress	≤ 23	16.34	5.59	0.03	133	0.974
	> 24	16.31	5.83			

*Significant at *p* ≤ 0.05.

#### Marital status

An independent –sample t-test was used to examine difference in the total level of empathy and subscales with respect to marital status. The result of study showed that there is statistical significant difference in the mean level of empathic concern subscale in scores between unmarried (M = 16.34, SD = 4.91) and married (M = 18.06, SD = 3.97, t (133) = -1.84, p = 0.044) (Table 6). These results reveled that married nursing interns scored higher in the empathic concern subscale than unmarried nursing interns. This mean that married nursing interns felt more sympathy and caring for others than unmarried nursing interns.

**Table 6:** Difference between Marital Status and IRI with Subscales

Variable	Marital status	Mean	SD	*t -Value*	*df*	*P-value*
Interpersonal Reactivity Index (IRI)	Unmarried	66.33	20.09	-1.20	133	0.231
	Married	70.82	14.38			
Fantasy	Unmarried	16.39	6.45	− 0.69	133	0.489
	Married	17.24	5.21			
Perspective-taking	Unmarried	17.51	5.62	− 0.90	133	0.369
	Married	18.47	4.39			
Empathic concern	Unmarried	16.34	4.91	-1.84	133	0.044*
	Married	18.06	3.97			
Personal distress	Unmarried	16.09	6.05	− 0.86	133	0.389
	Married	17.06	4.20			

*Significant at *p* ≤ 0.05.

#### Grade point average (GPA)

Participants in this study were divided into three groups according to their GPA. One way- ANOVA was used to examine difference in the level of empathy and subscales with respect to GPA. The result of study showed that there is no statistical significant difference at the p < 0.05 between different GPA points with total level of empathy and its subscale.

#### Clinical training area

Participants in this study were divided into thirteen groups according to their training areas. One way- ANOVA was used to examine difference in the level of empathy and subscales with respect to training areas. The result of study showed that there is no statistical significant difference at the p < 0.05 between different training areas with level of empathy, and subscales of fantasy, perspective-, empathic concern, and personal distress.

#### Preferred nursing as a Profession

An independent –sample t-test was used to examine difference in the level of total empathy and subscales with respect to preferred nursing as a profession. The result of study showed that there is statistical significant difference in the mean level of empathic concern subscale in scores between preferred nursing as a profession (M = 17.09, SD = 4.56) and not preferred nursing as a profession (M = 14.72, SD = 5.45, t (133) = -1.99, p = 0.049) (Table 7). These results reveled that nursing interns who preferred nursing as a profession had a statistically significant higher mean of subscale of empathic concern than nursing interns who did not.

**Table 7:** Difference between Preferred Nursing as a Profession and IRI with Subscales

Variable	Preferred Nursing	Mean	SD	*t -Value*	*df*	*P-value*
Interpersonal Reactivity Index	No	60.11	22.44	-1.78	133	0.076
	Yes	68.59	18.10			
Fantasy	No	14.28	7.05	-1.73	133	0.086
	Yes	16.96	5.96			
Perspective-taking	No	15.78	7.03	-1.69	133	0.092
	Yes	18.06	5.00			
Empathic concern	No	14.72	5.45	-1.99	133	0.049*
	Yes	17.09	4.56			
Personal distress	No	15.33	5.07	− 0.80	133	0.422
	Yes	16.49	5.73			

*Significant at *p* ≤ 0.05.

## Discussion

The purpose of this study was to examine the level of self-reported empathy among nursing interns. The results of this study showed that nursing interns showed a mean level of empathy of 67.46 (SD = 18.86). This result indicated that the nursing interns in the study have moderate levels of empathy overall. When compared to another recent Indian study conducted among nursing interns, this result implies a similar level of empathy [16]. In this regard, the literature shows that nursing interns have a significantly higher mean score of empathy than students in other professions [33, 34]. As stated by Giovanna, Chiara [35], among nursing professions, some specific skills are needed, such as knowing the basics of effective communication, using communication facilitation strategies, listening actively to the patient and understanding what it means to be in a demanding relationship. Nurses can develop tailored adaptation processes by using empathy and reflective thinking to deeply understand their patients’ feelings and lives. As a result, nursing care programs should assist nursing interns in communicating empathically with the patient as well as the interdisciplinary team.

The findings of this study demonstrated that there are no statistically significant changes in the mean level of empathy between different GPA points of nursing interns. This results is consistent with earlier studies [36, 37]. Other studies contradict the study’s finding that higher GPA students are more empathetic than lower GPA students [38, 39]. The difference in study results can be explained by nursing interns do not consider their GPA because they have completed their nursing education and their GPA will not be included in the internship period.

According to the findings of this study, there are no statistically significant differences in the mean level of empathy between different training areas. This result is similar to the findings of Ghaedi, Ashouri [40] which showed that nurses’ empathy levels are similar across hospital wards. However, the study result is not in line with most studies in others professions which highlight that medical students who chose general internal medicine and psychiatry residency as training area had the highest mean empathy ratings than physicians trained in anesthesia and surgery area [41, 42]. According to the author of this study, empathy is vital in any clinical environment, and nurses should be empathic with all patients regardless of clinical setting.

In this study, some sociodemographic characteristics that differ with empathy level were identified. Surprisingly, the current study reveals that there is a gender difference in empathy, with male students reporting a statistically significant higher mean of empathetic concerns and perspective-taking dimensions than female interns. This result contradicts most studies, which show that females are more empathic than males [33, 36, 38, 39, 43].

This divergent result might be explained by empathy, which is also defined from a cognitive component, which requires more complicated mental processes, allowing the individual to understand feelings in certain circumstances. This viewpoint is strongly related to theory of mind since it requires the ability to read and place ourselves in the mental and emotional place of another individual (perspective taking) [3]. The impact of gender on perspective taking may be explained by women being seen as more emotionally sensitive, while men are viewed as being less sensitive and more oriented towards a cognitive perspective. Another point of view, Persson and Hostler [44], perspective-taking mixed with self-affirmation improved empathic feelings toward feminists (as expressed by perspective-taking emotions). The effect on empathy was particularly significant among men who had previously had strong prejudices against feminists. As a result, male nursing interns may be able to reduce prejudice toward feminists by improving their empathic feelings. In addition, as the result of our study contradicts most of the previous studies, we suggest future studies to investigate more about empathy and gender through phenomenological study design.

The current study found that there is an age difference in empathy level, with younger nursing interns (≤ 23), reporting a statistically significant higher mean of perspective-taking dimension than older nursing interns (> 23). This result is consistent with the findings of Berduzco-Torres, Medina [38], who found that younger nursing students are more empathic than older nursing students and contradict studies of Ouzouni and Nakakis [43], Kesbakhi and Rohani [45] which highlight that older nursing students have higher empathy levels than younger students. The study’s findings can be justified by the fact that perspective taking declines with age. This justification was due to the cognitive requirements of taking another person’s perspective, which is reflected in evolutionary models of empathy whereby perspective taking is a higher-level process than experiencing empathic concern, and thus more vulnerable to decline with age process [46]. The results support this justification in general, with stronger perspective taking related with younger age. There is evidence of age-related declines in the cognitive ability to appropriately understand the emotions of others [47].

The current study found that marital status has an effect on empathy level, with married nursing interns reporting a statistically significant higher mean of empathic concern dimension than single nursing interns. This result is consistent with the findings of Sedaghati, Rohani [48] which found that married nurses had higher empathy concern scores, and contradicting a study by Kesbakhi and Rohani [45] that found single nursing students to be more empathic. Another study on oncology nurses in Turkey found that marital status had no effect on nurses’ empathetic concern [49]. According to the author, the conflicting results could be due to the low percentage of married students (25.2%) in this study, which has resulted in biased results.

### Implications for nursing practice

Relevant university decision makers, as well as nurse educators, should take seriously the current study’s finding that female, older, unmarried nursing interns appear to be less empathic than other nursing interns. This implies that they should engage in continuous reflection and educational activities as part of their clinical training as nursing interns in order to improve their empathic attitudes. For instance, The Empathy Educational Model (EEM) [50], was established specifically as an urgent requirement for empathy training with the increasing number of nursing schools, health organizations, community services and nursing homes. The EEM is based on the following training program steps with includes; enlightening nursing student with empathy world through using medical film and empathy experience, then, introduce nursing students to principle of empathy besides patient personality traits, after that, nursing students practice empathetic skills and later they need to evaluate and provide reflection report in their empathy skills. In addition, emphasis was giving for nursing students empathy training through situation teaching, task-based learning, video demonstration, and problem-based leaning. The EEM has been proved as an effective model that improve nursing empathy level which facilitate establishing nurse-patient relationships and enhance the mental health of both patients and nurse.

Furthermore, it is recommended teach and train nursing students on communication techniques, reflection skills, meditation and cultural aptitudes through the use of simulation techniques with mannequins or dealing with high-risk group can enhance the level of empathy. In addition, faculty and teaching staff play can use experiential learning techniques besides simulations and immersion in teaching and training different nurses’ course and workshop [51].

### Limitations

A number of limitations affect the interpretation of the study’s results. The use of only self-ratings is one limitation. It is commonly known that in self-rating empathy studies, individuals are likely to overestimate their empathy due to factors like social desirability. Other limitations include the fact that based on the nature of the study, the number of participnats consider small and was conducted at only one university, limiting the generalizability of the findings. Furthermore, because this is a cross-sectional analytic study, it is difficult to know the process and direction of the relationships that were identified, and the focus was on sociodemographic and academic associations.

## Conclusion

This is a study of self reported empathy among nursing interns in saudi Arabia. Perspective taking incresed with younger, male nursing interns, this reflects high cognitive flexibility with younger age nursing interns. Morover, the empathic concern incresed with married nuring interns who preferred nursing as a profession. Research results were discussed in details and recommendations were proposed in order to improve leve of empathy among nursing intern. For instance, the use of emotional educaitonal model, and teach and train nursing students on communication techniques, reflection skills, meditation and cultural aptitudes through the use of simulation techniques with mannequins can improve empathy level.

## Electronic supplementary material

Below is the link to the electronic supplementary material.

Supplementary Material 1

## Data Availability

The data that were generated and analyzed in this study are mostly included within the published article. However, source material and the raw datasets are available from the corresponding author upon request.

## Abbreviations

GPA: Grade Point Average
IRI: Interpersonal Reactivity Index
IAU: Imam Abdulrahman Bin Faisal University
SPSS: Statistical Package for the Social Sciences
EEM: Empathy Educational Model
